# Pre-clinical assessment of a water-in-fluorocarbon emulsion for the treatment of pulmonary vascular diseases

**DOI:** 10.1080/10717544.2019.1568621

**Published:** 2019-03-01

**Authors:** Scott K. Ferguson, David I. Pak, Justin L. Hopkins, Julie W. Harral, Katherine M. Redinius, Zoe Loomis, Kurt R. Stenmark, Mark A. Borden, Thies Schroeder, David C. Irwin

**Affiliations:** a Cardiovascular and Pulmonary Research Laboratory, Department of Medicine, University of Colorado Denver, Anschutz Medical Campus, Aurora, CO, USA;; b Department of Mechanical Engineering, University of Colorado, Boulder, CO, USA;; c Department of Biochemistry, Johannes-Gutenberg University, Mainz, Germany

**Keywords:** Pulmonary hypertension, pulmonary pressures, high altitude pulmonary edema, endothelin, ambrisentan, sodium nitrite, perfluorocarbon

## Abstract

Hypoxic pulmonary vasoconstriction (HPV) is a well-characterized vascular response to low oxygen pressures and is involved in life-threatening conditions such as high-altitude pulmonary edema (HAPE) and pulmonary arterial hypertension (PAH). While the efficacy of oral therapies can be affected by drug metabolism, or dose-limiting systemic toxicity, inhaled treatment via pressured metered dose inhalers (pMDI) may be an effective, nontoxic, practical alternative. We hypothesized that a stable water-in-perfluorooctyl bromide (PFOB) emulsion that provides solubility in common pMDI propellants, engineered for intrapulmonary delivery of pulmonary vasodilators, reverses HPV during acute hypoxia (HX). Male Sprague Dawley rats received two 10-min bouts of HX (13% O_2_) with 20 min of room air and drug application between exposures. Treatment groups: intrapulmonary delivery (PUL) of (1) saline; (2) ambrisentan in saline (0.1 mg/kg); (3) empty emulsion; (4) emulsion encapsulating ambrisentan or sodium nitrite (NaNO_2_) (0.1 and 0.5 mg/kg each); and intravenous (5) ambrisentan (0.1 mg/kg) or (6) NaNO_2_ (0.5 mg/kg). Neither PUL of saline or empty emulsion, nor infusions of drugs prevented pulmonary artery pressure (PAP) elevation (32.6 ± 3.2, 31.5 ± 1.2, 29.3 ± 1.8, and 30.2 ± 2.5 mmHg, respectively). In contrast, PUL of aqueous ambrisentan and both drug emulsions reduced PAP by 20–30% during HX, compared to controls. IL6 expression in bronchoalveolar lavage fluid and whole lung 24 h post-PUL did not differ among cohorts. We demonstrate proof-of-concept for delivering pulmonary vasodilators via aerosolized water-in-PFOB emulsion. This concept opens a potentially feasible and effective route of treating pulmonary vascular pathologies via pMDI.

## Introduction

Overshooting tightening, or constriction, of blood vessels of the lung, is a serious pathology that can be caused by various underlying factors: inspiration of air with a low oxygen tension (e.g. exposure to high altitude) leads to acute constriction of pulmonary arterial blood vessels, thus elevating pulmonary artery pressure (PAP), and forcing the right ventricle to pump against increased vascular resistance (Kylhammar & Radegran, 2017). The resulting hydrostatic pressure on the pulmonary microvasculature leads to increased fluid extravasation into the lung parenchyma, which can rapidly escalate to high altitude pulmonary edema (HAPE), the leading cause of altitude-related death (Bartsch & Gibbs, 2007).

Chronic vasoconstriction and the ensuing thickening of the pulmonary blood vessel walls is also the basis of congenital or acquired pulmonary arterial hypertension (PAH) and can lead to death via right-heart failure. Treatments aimed at lowering pulmonary pressures include vasodilators such as prostaglandin pathway agonists, phosphodiesterase inhibitors and other modulators of the nitric oxide (NO) signaling pathway, as well as, calcium channel blockers, glucocorticoids, and endothelin receptor antagonists (Galie et al., 2013; Swenson, 2016). These therapies are typically given orally and rely on the systemic circulation to deliver the drug to the pulmonary vasculature, which is a limited delivery tactic in this disease paradigm as many regions of the lung are underperfused due to the aforementioned vasoconstriction. On the other hand, the arterioles within the lung acini (i.e. units of several lung alveoli) are located near the blood-gas barrier and are actively involved in the control of pulmonary vascular resistance (Paddenberg et al., 2006, 2014), thus making them an attractive target for intrapulmonary therapies. Furthermore, the potential of reduced toxicity associated with a local (e.g. pulmonary) application, along with the possibility of the immediate onset of pharmacological activity, provides strong motivation to consider inhaled therapies as an alternative to oral forms of vasoactive drugs (Rashid et al., 2017).

However, the feasibility of reaching the distal pulmonary vasculature with an inhaled therapy depends on both the successful design of the aerosolized vehicle and the delivery device (Myrdal et al., 2014). In this regard, while a range of dry powder inhalers and nebulizers are currently available to deliver intrapulmonary therapies, pressurized metered dose inhalers (pMDI) combine several advantages over these other options: Compared to nebulizers, their compactness, lack of electrical components and ease of use facilitates their deployment as portable items during travel and by patients in remote locations. As opposed to other inhaled therapies like dry powder inhalers, pMDIs do not require the patient to maintain a constant and robust air flow while inhaling the drug, which is not optimal in disabled, weak, injured, or unconscious individuals, as well as in children and newborns.

Unfortunately, pMDIs rely on the use of propellants that generate constant output pressure independent of filing status, and the most commonly used propellants (i.e. HFA 134a or HFA 227) have extremely low water solubility (2200 and 610 ppm, respectively) (Myrdal et al., 2014) making it difficult to deliver adequate amounts of a chosen therapeutic. However, with water-in-fluorocarbon technology, it is possible to encapsulate an active pharmaceutical ingredient (API) within an emulsion, thus affording compatibility with pMDI propellants and enabling enhanced delivery of API to distal regions of the lung without adherence to the upper airways (Courrier et al., 2004). While these therapeutic technologies seem promising, to our knowledge there have been no investigations into the efficacy of a water-in-fluorocarbon based intrapulmonary therapeutic aimed at the treatment of pulmonary vascular disease and the associated elevations in PAP.

Thus, the purpose of this investigation was to test whether an inhaled treatment by utilizing a water-in-fluorocarbon emulsion can attenuate hypoxic pulmonary vasoconstriction (HPV) in rats exposed to hypoxia. We tested the hypothesis that, relative to an empty emulsion, a water-in-fluorocarbon emulsion containing either the endothelin receptor type-A antagonist ambrisentan or the NO donor sodium nitrite (NaNO_2_) would significantly attenuate the HPV response initiated by simulated altitude. Results from this investigation will provide the crucial evidence needed to develop a therapeutic further using a water-in-fluorocarbon reverse emulsion that is compatible with common pMDI propellants.

## Materials and methods

### Animals and ethical approval

Adult male Sprague-Dawley rats (*n* = 49) were obtained from Charles River (Wilmington, MA). Before any animal studies, all experimental protocols were reviewed and approved by the Institutional Animal Care and Use Committee at the University of Colorado Denver, Anschutz Medical Campus. Animals were allowed access to food and water *ad libitum* and were kept on a 12-h day-night cycle.

### Emulsion preparation

Emulsions were prepared using di-morpholino-4-yl-phosphinic acid 12, 12, 13, 13, 14, 14, 15, 15, 16, 16, 17, 17, 18, 18, 19, 19, 19-heptadecafluoro-nonadecyl ester (F8H11DMP) (PharmAgra Labs; Brevard, NC) as the surfactant, and the bulk medium phase was composed of PFOB (Fluoromed, L.P.; Round Rock, TX). The water phase consisted of phosphate-buffered saline (PBS; 0.1 M NaCl, pH 8), and either ambrisentan (Duke Small Molecule Synthesis Facility; Durham, NC) or NaNO_2_ (Sigma-Aldrich; St. Louis, MO) dissolved at the solubility limit (ambrisentan: 150 mg/ml, NaNO_2_: 820 mg/ml) in PBS.

The F8H11DMP was solubilized in PFOB at a concentration of 1.5% w/v (mg/mL). Next, the PBS (or PBS solution with the API) was added to the surfactant/PFOB solution in a volume ratio 1:9 v/v which has previously shown the best stability (Butz et al., 2002). The mixture was then emulsified using a D650 Amalgamator (TPC Advanced Technologies; City of Industry, CA) at a rate of 4400 RPM at 40-second intervals. Intrapulmonary treatments were delivered through an endotracheal tube placed in the rats using a Microsprayer® attached to the FMJ-250 syringe (Penn-Century; Wyndmoor, PA).

### Study drugs

Ambrisentan is an endothelin-1 receptor A antagonist that blocks the vasoconstrictive response in blood vessels from the endothelin in the blood (Galie et al., 2013). Additionally, we have tested the use of NaNO_2_, a NO reservoir that has shown efficacy in acting as a pulmonary vasodilator (Zuckerbraun et al., 2010).

### Experimental protocols

To study the effectiveness of intrapulmonary delivery of the API, ambrisentan and NaNO_2_, rats were randomly assigned to the following nine groups (*n* = 5–8 per group) (1) saline; (2) aqueous ambrisentan (i.e dissolved in saline) (0.1 mg/kg); (3) empty emulsion (without an API); (4) high dose ambrisentan emulsion (0.5 mg/kg); (5) low dose ambrisentan emulsion (0.1 mg/kg); (6) high dose NaNO_2_ (0.5 mg/kg); (7) low dose NaNO_2_ (0.1 mg/kg); (8) intravenous (IV) infusion of ambrisentan (0.1 mg/kg); and (9) IV infusion of NaNO_2_ (0.5 mg/kg). The doses of ambrisentan were calculated relative to human doses used in the clinical safety and efficacy studies for pulmonary hypertension (PH) (Galie et al., 2008), and doses of NaNO_2_ were chosen on the basis of previously published data for IV infusion of NaNO_2_ in conscious rats (Ferguson et al., 2016).

## Surgical procedures and hemodynamic instrumentation

Rats were anesthetized by intramuscular injection with a ketamine/xylazine mixture (75 and 6 mg/kg, respectively). The ventral neck was shaved, and a 2-cm incision was made in the right ventral neck to isolate the jugular vein and right carotid artery via blunt dissection. A polyethylene (PE-50) catheter was introduced into the carotid artery to measure systemic blood pressure. A polyvinyl (PV-1) catheter was connected to pressure transducers and monitored continuously with the MP150 data acquisition system (BioPAC Systems; Goleta, CA) inserted into the jugular vein and threaded through the right atrium, right ventricle, and into the lumen of the main pulmonary artery. The location of the tip of the catheter is identified and positioned by the characteristic shape of the pressure waveforms, as well as the change in diastolic pressure between RV and PA. Both systemic and pulmonary fluid-filled catheters were connected to pressure transducers and monitored continuously with the MP150 data acquisition system as previously mentioned above. Blood pressures were recorded every two minutes for data analysis. For the IV treated groups, an additional PV-1 catheter was inserted into the femoral vein to avoid interrupting hemodynamic measurements while administering treatment.

**Figure 1.**
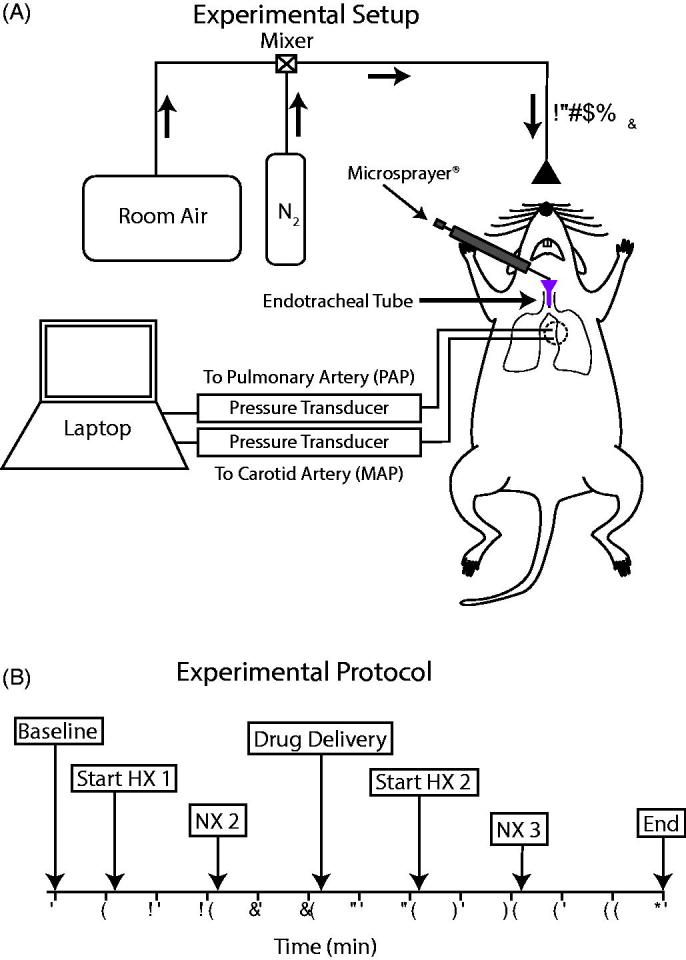
Experimental design. (A) Schematic of the *in vivo* experimental setup. Hypoxic conditions were induced by mixing room air with N_2_ to deliver 13% O_2_. The emulsion was administered to the lungs via the endotracheal tube using a Microsprayer®. Mean pulmonary arterial pressure (PAP) and mean arterial pressure (MAP) were measured using fluid-filled indwelling catheters connected to pressure transducers and recorded with a laptop using the BioPac MP150 system. (B) Experimental protocol indicating time points for normoxia (NX), hypoxia (HX), and drug delivery throughout the experiment.

After completion of catheter placements and instrumentation for hemodynamic measurements, a 1-cm incision was made above the trachea, and the trachea was isolated by blunt dissection. A tracheotomy was performed ∼4-mm above the carina and an endotracheal tube specially designed for rats was inserted such that the end of the tube was placed roughly 2-mm distal from the carina. This placement allowed the tip of a Microsprayer^®^ to protrude ∼1-mm from the tracheal tube for intrapulmonary treatment to both right and left lung periphery (Figure 1(A)).

## Microsprayer^®^ distribution of perfluorocarbon by PET scan

To observe the distribution of the Microsprayer^®^ aerosol within the rat lung in our model system, a radiolabeled fluorinated hydrocarbon created from PFOB via a one-step substitution nucleophilic 2nd order reaction. The bromine was replaced with an ^18 ^F for a PET scan analysis to take sagittal and coronal images of the lung distribution over 3 min.

## Evaluation of intrapulmonary therapy

Following the instrumentation and tracheotomy, anesthetized rats were placed in a specially designed Plexiglas box built to accommodate exteriorized catheters for the completion of the study protocol (Figure 1(B)). Baseline mean PAP and mean arterial pressure (MAP) were recorded. Next, the rats were switched from breathing room air to a hypoxic gas mixture for 10 min (13% O_2_ via room air/nitrogen dilution; Figure 1(A)). This model previously demonstrated a robust HPV-mediated response that significantly raised pulmonary arterial pressures (Butz et al., 2002).

This first hypoxic challenge was followed by 20 min of room air breathing. The purpose of this initial bout of hypoxic air breathing was to confirm that each animal had an intact HPV-mediated rise in PAP. During the interim period of room air breathing, rats were allowed to recover from hypoxia for 10 min before treatment administration. Treatments were delivered as a 100 µL bolus aerosol immediately after emulsification or as a 0.5 mL IV infusion over 30 sec.

Following treatment, rats remained in breathing room air for an additional 10 min to allow drug absorption/activation before being exposed to a second 10-minute hypoxic challenge. Following the second hypoxic challenge, rats returned to breathing room air, and blood pressures were monitored for an additional 10 min before being humanely euthanized with an intravenous overdose of sodium pentobarbital.

### Observations of physiological changes after treatment

#### *In vivo* toxicity study

A separate group of rats was studied to observe if either the empty emulsion or emulsion encapsulating an API induced lung inflammation indicative of toxicity related side effects. Rats were randomized into 3 treatment groups (*n* = 4 per group): (1) saline; (2) ambrisentan emulsion (5 mg/kg); and (3) sodium nitrite emulsion (5 mg/kg). These high doses (10× the effective dose) were chosen on the basis of eliciting any type of inflammatory response from an assumed relatively inert compound. Rats were anesthetized with intramuscular injection of ketamine/xylazine (75 and 5 mg/kg) and inhaled isofluorane (5% in room air at 1 L/min). While anesthetized, a tracheal cannula was inserted between breaths, and a 100-µL bolus aerosol was administered. Rats were monitored while recovering from the anesthesia and hemodynamic data were collected 24-h post-treatment. Left ventricular pressure was directly measured using a 1.9 French Pressure-Volume catheter (FTE-1912B-6018) (Transonic Systems Inc.; Ithaca, NY), and data were recorded continuously with LabScribe (iWorx; Dover, NH).

#### Bronchoalveolar lavage (BAL) for ELISA and cell counting

To understand potential local toxicity, alveolar lung cells were collected from these rats by bronchoalveolar lavage (BAL). The lavage was repeated three times with 5 mL of cooled PBS for cell extraction. After the first lavage, 1 mL of the lavage fluid was placed in a tube and centrifuged at 13,000 rpm for 5 min and frozen in liquid nitrogen for IL-6 ELISA (Abcam; Cambridge, UK, England).

The remaining fluid from the lavages was collected in a conical tube and centrifuged at 1100 rpm for 10 min. The supernatant was disposed, and cells were resuspended in 1 mL of sterile PBS. A 100 µL sample of the cell suspension was centrifuged onto a slide at 800 rpm for 3 min, fixed with methanol, and stained with hematoxylin to quantify the number of alveolar macrophages. Cell counts were analyzed using Image J (Naional Institutes of Health, Bethesda, MD, USA) where randomized sample slices from the slide and were used to estimate the average macrophage count in each rat and compared across treatment groups.

#### Western blot analysis and qRT-PCR for IL-6 expression

As a method of verification for IL-6 expression, protein lysates were prepared from whole lung tissue for standard western blot analysis. Samples were run on pre-cast 18 well 15% criterion TRIS-HCl gels (Bio-Rad Laboratories; Hercules, CA) and probed using mouse anti-IL-6 (1:1000, ab9324) and mouse anti-Beta-actin (1:1000, ab8229). Image J was used to determine the density of the IL-6 and Beta-actin protein bands, and IL-6 to Beta-actin ratios were used to compare IL-6 expression across treatment groups.

Real-time PCR was conducted using whole lung tissue to probe for IL-6 and Beta-actin protein expression. RNA was extracted from the tissue using Qiagen RNeasy Plus Mini Kit (Qiagen; Hilden, Germany) and converted to cDNA by PCR, and the qRT-PCR was completed using the QuantiFast SYBR Green PCR Kit (Qiagen; Hilden, Germany).

#### Histological analysis

The left lung was inflated with 8 mL of a 5% SeaPlaque^®^ Agarose solution (Lonza; Basel, Switzerland) then cooled and fixed in formalin. Following formalin fixation, the left lung was paraffin-embedded, sliced in 1 µm sections and stained with hematoxylin and eosin. Lung slides were imaged for histopathology analysis using the Aperio Versa 8 (Leica Biosystems; Buffalo Grove, IL).

### Statistical analysis

All values are reported as the mean ± standard error of the mean (SEM). Statistical comparisons for data measurements were completed using one-way analysis of variance (Kwon et al., 2015) with the Tukey correction for multiple comparisons. All statistical analyses were performed using the GraphPad Prism 7 statistical software package (Graphpad Software, Inc; La Jolla, CA) with statistical significance set at *p* < .05.

## Results

### Emulsion characterization

**Figure 2.**
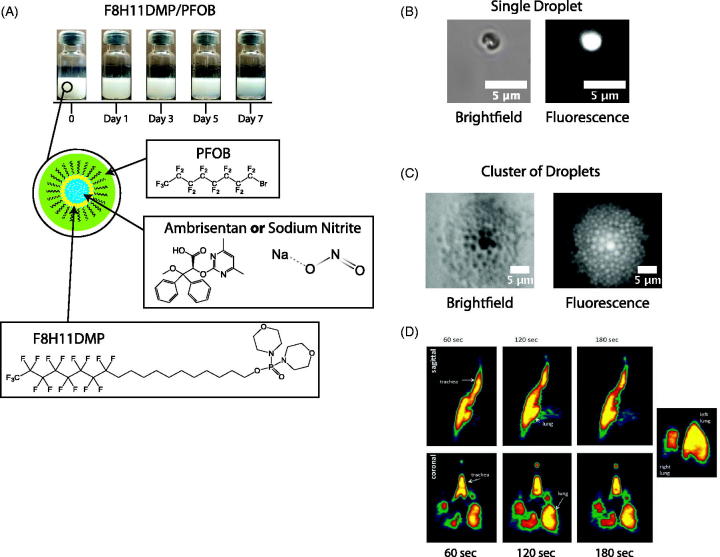
Emulsion characterization. (A) Sequential images of the emulsions over a 7-day period. Phase separation occurred after ∼3 days. Schematics of the formulation with the chemical structures for PFOB, F8H11DMP, ambrisentan, and sodium nitrite (NaNO_2_) are presented for the composition of the emulsion droplets. (B,C) Brightfield and fluorescent microscopy images of the emulsion for a single and cluster of droplets, respectively, and are <5 µm in diameter. (D) PET scan time sequenced images of the distribution of the aerosol in the rat lungs using the radiolabeled ^18^F.

To encapsulate an API in the emulsion for delivery via a pMDI, droplet stability is imperative. Using the semi-fluorinated surfactant, F8H11DMP, phase separation was not evident until three days after emulsification and did not significantly progress further between days 3 and 7 (Figure 2(A)). Additionally, to confirm that we had created a water-in-fluorocarbon composition, fluorescein isothiocyanate-dextran was added to the water phase, and the droplets were observed using fluorescent microscopy. Our observations confirmed a water-in-fluorocarbon composition, and droplet diameters ranged from 1 to 5 µm (Figure 2(B,C)).

#### Microsprayer^®^ distribution of perfluorocarbon by PET scan

The radiolabeled perfluorocarbon provided a PET scan with color graded concentrations within the lung. As expected, continuous scanning over a 3-min period confirmed that dispersion from the Microsprayer^®^ was distributed throughout the lung (Figure 2(D)). The highest concentrations (red) lined the wall of the bronchial tree, but high concentrations (yellow) were noted throughout the lung including distal regions (Figure 2(D)).

### *In vivo* intrapulmonary drug delivery characterization

#### Baseline recordings

**Figure 3.**
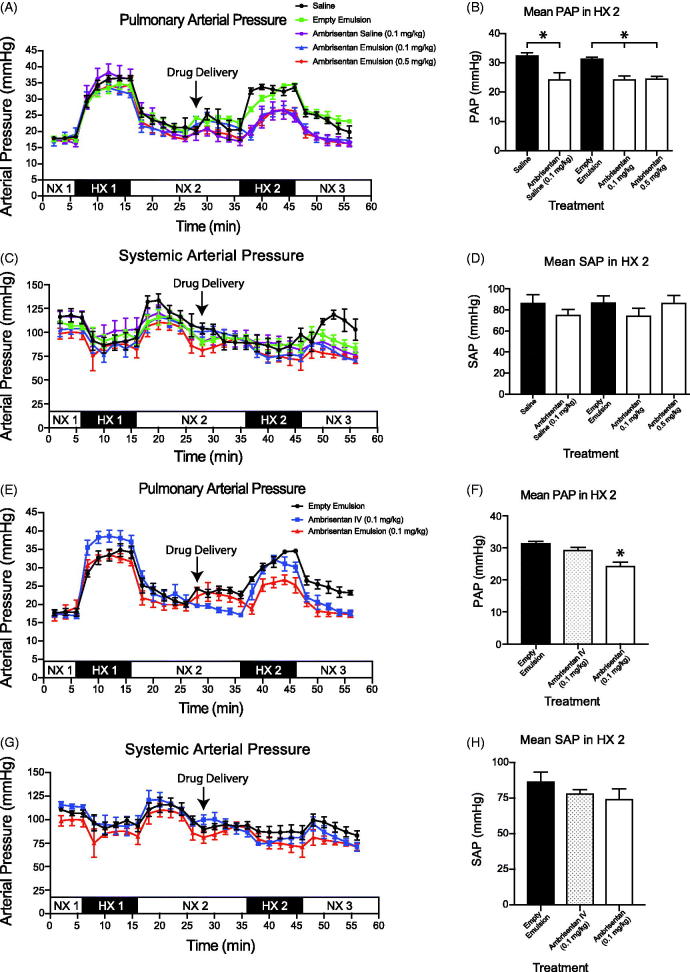
*In vivo* evaluation of the delivery of ambrisentan (Doty et al.,^2017^). Mean pulmonary artery pressure (PAP) of intrapulmonary treatment groups. (C, D) Mean arterial pressure (MAP) of intrapulmonary treatment groups. Measurements were recorded at 2 min intervals and represent mean ± SEM. Bar graphs represent the average pressure ± SEM over the 10 min time span of the second hypoxic challenge (HX 2). Saline (•); Empty Emulsion (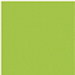); Ambrisentan Saline (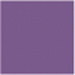); Ambrisentan Emulsion (0.1 mg/kg) (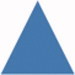); Ambrisentan Emulsion (0.5 mg/kg) (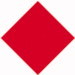); NX: normoxic; HX: hypoxic. *p < .05 (Haase et al.,^2010^). Mean pulmonary artery pressure (PAP) of intravenous infusion and ambrisentan emulsion (Isaac et al., 2006). Mean arterial pressure (MAP) of intravenous infusion and ambrisentan emulsion. Empty Emulsion (•); Ambrisentan IV (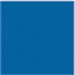); Ambrisentan Emulsion (0.1 mg/kg) (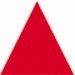).

Baseline mean PAP and MAP obtained in NX1 period was similar among all rats and confirmed normal healthy animals across cohorts (Figure 3(A)). We observed the expected percent increase in PAP (94.7 ± 4.3%) and fall in MAP (18.5 ± 3.1%) after challenging rats with hypoxic air. Both PAP and MAP returned to baseline values within 10 min once rats were returned to room air breathing.

#### Intrapulmonary drug administration

All rats tolerated the intrapulmonary delivery of the emulsions administered via the Microsprayer^®^ through the endotracheal tube in the NX2 period (Figure 3). We observed no significant changes in either PAP, MAP (Figure 3), breathing rate, or heart rates (data not shown) within 10 min after administration and prior to the second (HX2) hypoxic challenge.

#### Pulmonary artery pressures

During the HX2 period, intrapulmonary administration of either saline or empty emulsion did not affect PAP when compared within subject groups (HX1 vs. HX2) or between (HX2 saline vs. HX2 empty emulsion) cohorts (saline: 32.6 ± 3.2 mmHg; empty emulsion: 31.5 ± 1.2 mmHg).

**Figure 4.**
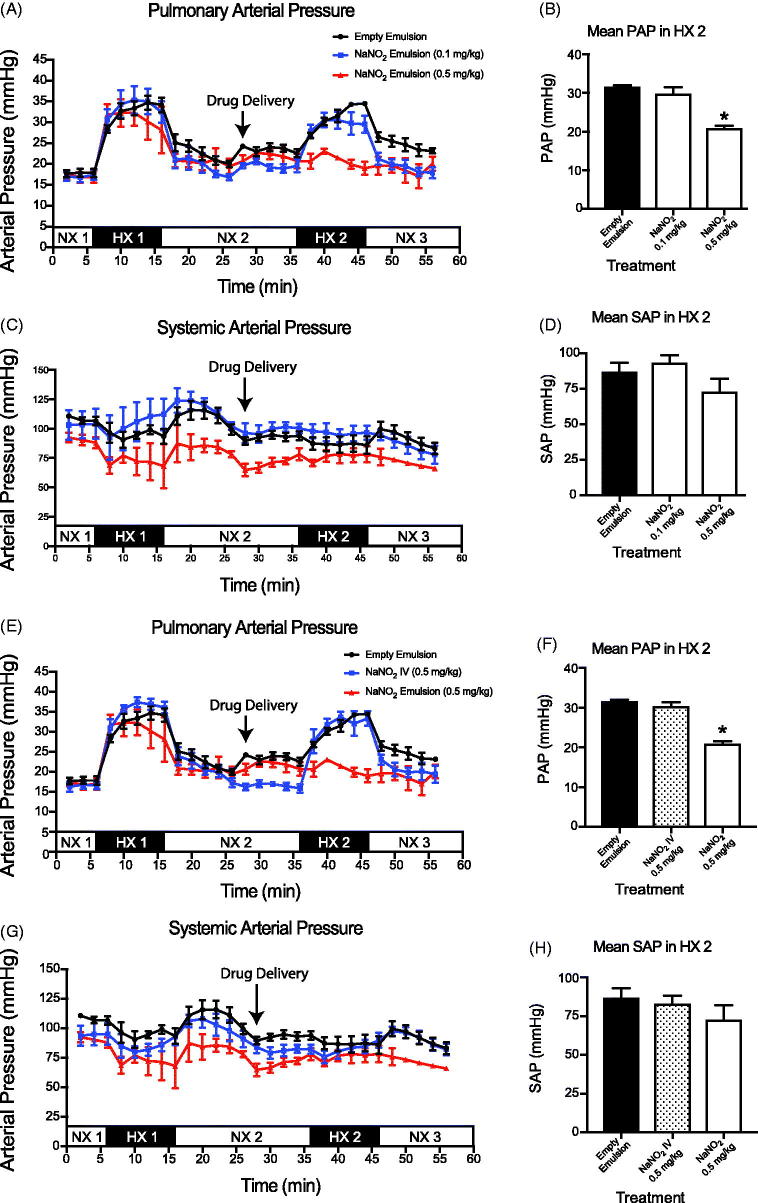
*In vivo* evaluation of the delivery of sodium nitrite (NaNO_2_) (Doty et al.,^2017^). Mean pulmonary artery pressure (PAP) of intrapulmonary treatment groups. (C, D) Mean arterial pressure (MAP) of intrapulmonary treatment groups. Measurements were recorded at 2 min intervals and represent mean ± SEM. Bar graphs represent the average pressure ± SEM over the 10 min time span of the second bout of hypoxia (HX 2). Empty Emulsion (•); NaNO_2_ Emulsion (0.1 mg/kg) (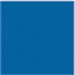); NaNO_2_ Emulsion (0.5 mg/kg) (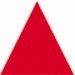); NX - normoxic; HX - hypoxic. *p < .05 (Haase et al.,^2010^). Mean pulmonary artery pressure (PAP) of intravenous infusion and NaNO_2_ emulsion (Isaac et al.,^2006^). Mean arterial pressure (MAP) of intravenous infusion and NaNO_2_ emulsion. Empty Emulsion (•); NaNO_2_ IV (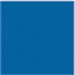) NaNO_2_ Emulsion (0.5 mg/kg) (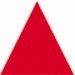).

In comparison to the saline and empty emulsion treatment cohorts, intrapulmonary delivery of ambrisentan (0.1 and 0.5 mg/kg) encapsulated in the emulsion attenuated the rise in PAP by 24% (Figure 3(A,B)). Intrapulmonary delivery of aqueous ambrisentan was as effective in attenuating the PAP as both doses of encapsulated ambrisentan during the HX2 period (Figure 3(A,B)). For the NaNO_2_ groups, the high dose NaNO_2_ (0.5 mg/kg) significantly reduced PAP (20.8 ± 1.1 mmHg) during the second hypoxic period (HX2). However, these results were not observed for the low dose NaNO_2_ (29.6 ± 3.7 mmHg, Figure 4(A,B)).

Intravenous therapies provide the quickest and most bioavailable route to alter pulmonary vascular tone. Thus, as an index to compare intrapulmonary delivery of the ambrisentan and NaNO_2_ emulsions, we treated rats with IV infusion of either ambrisentan or NaNO_2_ at the lowest effective dose that we observed for either API (i.e. 0.1 mg/kg ambrisentan or 0.5 mg/kg NaNO_2_). In contrast to intrapulmonary delivery, neither IV infusion of ambrisentan nor NaNO_2_ significantly reduced the PAP during the HX2 challenge when compared to the vehicle controls (Figures 3 and 4(E,F)).

#### Mean arterial blood pressures (MAP)

Neither intrapulmonary delivery nor IV infusion of ambrisentan significantly altered the MAP compared to rats that received intrapulmonary saline or empty emulsion during the HX2 period (Figures 3 and 4(C,D,G,H)). Similarly, neither intrapulmonary delivery nor IV infusion of NaNO_2_ altered MAP.

### Observations of physiological changes after treatment

**Figure 5.**
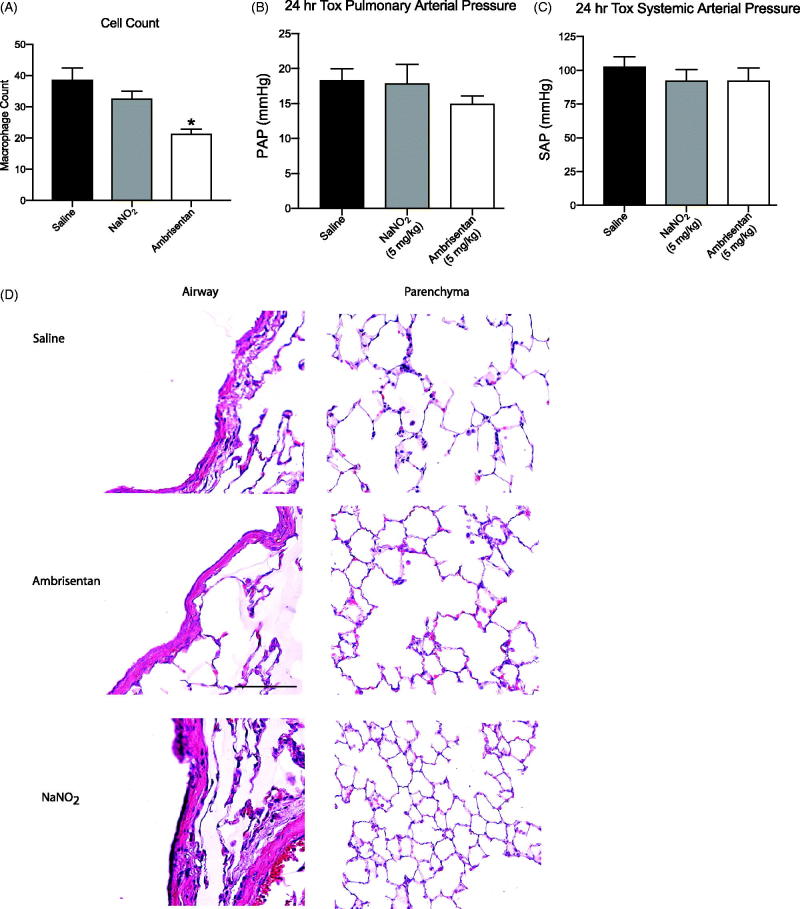
Observations of physiological changes after dosing. (A) Macrophage count per frame *p < .05. (B) PAP 24 h after administered dose. (C) MAP 24 h after administered dose. (D) Histopathology panel of H&E stained lungs harvested and inflated 24 h after administered dose for saline, ambrisentan emulsion (5 mg/kg), and NaNO_2_ emulsion (5 mg/kg).

The analysis of BAL fluid revealed that macrophage cell concentrations were significantly lower (*p* < .05) following ambrisentan treatment compared to rats that received aerosolized saline and NaNO_2_ emulsion (Figure 5(A)). However, ELISA revealed no differences in lung tissue IL-6 expression between rats exposed to aerosolized saline compared to rats exposed to either aqueous ambrisentan or NaNO_2_ (data not shown). This is supported upon further analysis of the western blot and qRT-PCR, and no significant changes in IL-6 expression were observed. There were no obvious anatomical changes related to the airways of the lungs (large gaps in the lung parenchyma are artifacts of lung inflation procedures, Figure 5(D)).

## Discussion

The principal original finding of this investigation is that a pulmonary vasoactive drug encapsulated in a fluorocarbon amphiphile micelle yields a stable formulation for intrapulmonary delivery of the endothelin receptor type A antagonist ambrisentan, and shows efficacy *in vivo*. Specifically, and consistent with our original hypothesis, intrapulmonary delivery of both ambrisentan and NaNO_2_ via the water-in-fluorocarbon emulsion significantly attenuated the HPV response to simulated altitude. Crucially, the F8H11DMP emulsion demonstrated sufficient stability for *in vivo* application, suggesting this technology could be developed into a practical and potentially safe means of treating pulmonary vascular diseases.

### Emulsion characterization

The successful synthesis and application of a water-in-fluorocarbon emulsion for intrapulmonary drug delivery systems depend largely on stability, homogeneity, and droplet size (Prieve & Russel, 1988; Courrier et al., 2004; Myrdal et al., 2014). The F8H11DMP emulsion demonstrated sufficient stability for *in vivo* application likely due, at least in part, to the surface forces that potentiate coalescence (Vervaet & Byron, 1999). For water-in-oil emulsions, the main attractive force driving coalescence is the van der Waals surface force, which is proportional to the Hamaker constant during droplet-droplet separations. By matching the dielectric constants and index of refraction between the bulk median PFOB and surfactant F8H11DMP, the resulting Hamaker constant is reduced to approximately zero, leading to the observed emulsion stability. These results are crucial to facilitate further development of this technology and promote its use as a potential therapeutic.

### In vivo experimentation and practical implications

It is well recognized that the use of saline as a pMDI excipient exhibits poor solubility with HFA propellants (Swenson & Bartsch, 2012). The aim of administering ambrisentan at equimolar amounts to the lung, relative to the aqueous drug solution, was to identify any physiological effects that may be caused by the addition of the formulation. We observed no differences between cohorts that received ambrisentan in the emulsion when compared to aqueous ambrisentan, thus suggesting that the emulsion itself does not negatively interfere with drug delivery.

Of note, the dose of ambrisentan used in the present investigation was approximately half of the human equivalent oral dose administered to treat pulmonary hypertension. Despite the smaller dose, the hypoxia-induced rise in PAP from the HPV response was significantly attenuated following treatment. While it is unclear whether intrapulmonary delivery of lower doses of ambrisentan would effectively treat human pulmonary hypertension, these data support the use of an intrapulmonary delivery modality for the ‘advanced’ treatment of PH. ‘Advanced’ treatment is directed at improving symptoms of PH and increasing quality of life for the patient and includes prostacyclin agonists, endothelin receptor antagonists, nitric oxide and cGMP enhancers. ‘Primary’ treatment, on the other hand, is directed at targeting the cause of PH, such as improving underlying cardiac issues in PH group 2 patients, or providing supplemental oxygen to PH patients categorized in group 3 PH (Hopkins & Rubin, 2018). It is also possible that enhanced intrapulmonary drug delivery was facilitated by the F8H11DMP surfactant itself, which has shown affinity to the SP-B and SP-C molecules in the lung surfactant *in vitro* (Nakahara et al., 2010).

One unexpected finding of the present investigation was that the high dose NaNO_2_ emulsion effectively attenuated the induced rise in PAP, while the low dose NaNO_2_ emulsion did not significantly affect the HPV response. Since the reduction of NO_2_ to NO is crucial for the vasoactive effects of NaNO_2_ treatment, it is possible that the amount of NO generated from the low-dose of NaNO_2_ was merely insufficient to cause a significant physiological effect. Considering that elevated blood plasma levels of NO_2_ have been linked with improvements in cardiovascular control (Lundberg et al., 2008; Ferguson et al., 2013), future investigations into the effects of PFC delivered NO_2_ on systemic NO_2_ concentrations, and their effect on peripheral vascular and metabolic function are warranted.

Contrary to the effective use of the inhalational therapy, HPV was not effectively reversed by IV infusion of either ambrisentan or NaNO_2_ despite the equimolar doses in both treatment modalities. It may be assumed that drugs delivered via the pulmonary blood flow during the HPV response are not efficiently transported to the sites of constricted pulmonary arteries due to pulmonary blood shunting upstream of the alveoli (Tremblay et al., 2015). Conversely, intrapulmonary delivery may reach the smooth muscle cells more efficiently, thus achieving higher bioavailability than IV administration. While the reasons for the observed differences in efficacy between IV and intrapulmonary delivery modalities remain speculative, intrapulmonary drug delivery appears to provide a promising and more effective means to target pulmonary vascular disease than IV drug delivery.

### Physiological changes after treatment

Although water-in-fluorocarbon emulsions with fluorocarbon chains appear to be mostly biologically inert (Courrier et al., 2004), the combination of the emulsion encapsulating the drugs may cause some irritation of the pulmonary tissues. To address this concern, we quantified alveolar macrophages as well as IL-6 concentration and expression in BAL and whole lung 24 h after intrapulmonary administration of our API in the emulsion. IL-6 is expressed robustly in acute and chronic inflammatory processes of the lung (Rubini, 2013). The endothelin receptor blockade has demonstrated anti-inflammatory effects in macrophages (Gerlach et al., 2015), and interestingly the alveolar macrophage cell counts were significantly lowered after rats were treated with ambrisentan. However, analysis of IL-6 expression in the BAL and whole lung did not reveal any significant changes in protein expression.

Supporting these observations, ELISA, western blot analysis, and qRT-PCR revealed no significant difference in IL-6 expression among the three treatment groups. The histology panels also revealed no anatomical treatment-related changes between groups. This suggests that intrapulmonary delivery of drug emulsion did not cause inflammation; although we acknowledge that a more robust analysis including cytokine panels and histopathology for other inflammatory markers will be needed to rule out potential lung toxicity induced from the emulsion entirely. Nevertheless, these data support the hypothesis of the inherent safety of water-in-fluorocarbon formulations for intrapulmonary drug delivery.

### Clinical significance

Inhaled therapy targeting HPV provides a more advantageous route of administration than intravenous application due to several key factors. For example, inhalers do not require medically trained assistance for administration, offer an immediate onset of pharmacological activity, and omit gastrointestinal side effects (Galie et al., 2008). On the other hand, devices such as dry powder inhalers offer certain advantages over pMDIs or other inhalers, such as higher dose-per-actuation. However, pMDIs also do not require a full exhalation before actuation or a sustained inflow of air during inhalation, making intrapulmonary treatment easier, and even possible, to individuals or patients incapable of handling a dry-powder inhaler (Myrdal et al., 2014). For those in need of treatment who are unable to fully cooperate in receiving treatment (disabled, weak, injured or unconscious individuals, newborns, and small children), pMDIs are often the only existing alternative to dry powder inhalers to deliver treatments to the pulmonary system (Inhaler Error Steering et al., 2013). The formulation described in this work supports the feasibility of administering therapy through a pMDI with up to a 1000-fold increase in solubility with the pMDI propellants when compared to saline alone (Butz et al., 2002; Myrdal et al., 2014). At the same time, the F8H11DMP fluoroamphiphile used in this study has shown a promising safety profile, mostly due to its relatively high fluorocarbon chain length that prevents biochemical interaction with cellular compounds (Courrier et al., 2004).

With regards to emergency medicine, inhaled delivery of pulmonary vasodilators could provide repeated, expedient, and more effective treatment with potentially fewer side effects to individuals suffering from HAPE, especially if the person is weak or unconscious. The expedited drug delivery afforded by pMDIs is crucial when considering that HAPE has a rapid onset and yields an estimated mortality rate of 50% if left untreated (Swenson & Bartsch, 2012). Currently, oral administration of calcium channel blockers and phosphodiesterase inhibitors are considered the gold standard treatment of HAPE (Swenson & Bartsch, 2012). For those who travel to, or work in, areas of high elevation, slow drug bioavailability from oral treatment may not allow a person to remain ambulatory during descent, substantially increasing the chance of poor patient prognosis and increasing the risk to those involved in a rescue attempt. Thus, these results provide compelling evidence to support the feasibility of water-soluble drug delivery via a water-in-fluorocarbon formulation in a pMDI to treat pulmonary vascular, as well as other pulmonary and systemic diseases.

## Conclusions

This is the first *in vivo* proof-of-principle investigation into the development and efficacy of a water-in-fluorocarbon based intrapulmonary therapeutic aimed at pulmonary vascular pathologies. Consistent with our original hypothesis, intrapulmonary delivery of ambrisentan and NaNO_2_ attenuated the increase in PAP during acute exposure to hypoxia when compared to control groups. With the precautionary note that sufficient dose rates need to be achieved for human application, these data offer important evidence in support of the pre-clinical to the clinical transition of this drug delivery technology and suggest a potential means by which to treat pulmonary (and potentially systemic) pathologies using an intrapulmonary based therapy.
